# Serum hepatitis B virus RNA levels as a predictor of HBeAg seroconversion during treatment with peginterferon alfa-2a

**DOI:** 10.1186/s12985-019-1152-6

**Published:** 2019-05-07

**Authors:** Wen Jia, Men Qi Zhu, Xun Qi, Ting Wang, Xiao Wen, Pei Dong Chen, Qing Qi Fan, Wen-Hong Zhang, Ji Ming Zhang

**Affiliations:** 1 Department of Infectious Diseases, Jing’An District Centre Hospital of Shanghai, Fudan University, Shanghai, China; 2 Department of Infectious Diseases, Huashan Hospital, Fudan University, Room 510, Building 5, 12 Middle Wulumuqi Road, Shanghai, China

**Keywords:** HBV RNA, Hepatitis B e antigens, Hepatitis B, Chronic

## Abstract

**Background:**

Hepatitis B e antigen (HBeAg) seroconversion represents an endpoint of treatment of chronic hepatitis B virus (HBV) infections.

**Methods:**

We have studied whether levels of serum hepatitis B virus ribonucleic acid (HBV RNA) during pegylated interferon alfa-2a treatment might be helpful for predicting HBeAg seroconversion. 61 HBeAg-positive chronic hepatitis B (CHB) patients treated with pegylated interferon alfa-2a alone or in combination with adefovir (10 mg/day) for 48 weeks were included in this retrospective analysis. Response was defined as HBeAg seroconversion at 24 weeks posttreatment. Receiver operating characteristic analyses were used to identify baseline and on-treatment HBV RNA levels associated with response.

**Results:**

Twenty-two of 61 (36.1%) patients achieved a response. Baseline HBV RNA levels were lower in responders than in nonresponders (4.55 ± 1.19 and 5.90 ± 1.13 copies/mL, respectively, *P* = 0.001). Baseline HBV RNA cut off level (200,000 copies/mL) provided a positive predictive value (PPV) of 56.0% and a negative predictive value (NPV) of 77.8%. HBV RNA level (3000 copies/mL) at week 12 provide a PPV of 75.0% and a NPV of 82.8%. Moreover, HBeAg seroconversion rates at 24 weeks posttreatment were significantly higher in patients with HBV RNA ≤ 200,000 copies/mL at baseline and HBV RNA ≤ 3000 copies/mL at week 12 (92.9%) versus others (12.5%) (All *P* < 0.05).

**Conclusions:**

In Conclusions, serum HBV RNA levels may serve as a novel tool for prediction of HBeAg seroconversion during therapy with pegylated interferon alfa-2a in HBeAg-positive CHB patients.

**Electronic supplementary material:**

The online version of this article (10.1186/s12985-019-1152-6) contains supplementary material, which is available to authorized users.

## Background

Although effective vaccine against hepatitis B virus infection have been available for more than three decades, HBV infection remains a global health problem [1, 2]. Patients with chronic hepatitis B (CHB) have an increased risk of developing cirrhosis, hepatic decompensation, and hepatocellular carcinoma (HCC), which result in about 1 million deaths per year [3].

Antiviral treatment is effective in halting progression of CHB in many patients. Two classes of antiviral agents are available: nucleos(t)ide analogues (NA), such as entecavir, which inhibit the viral polymerase and interfere with viral replication, and interferon, including conventional and pegylated forms, which has antiviral and immunomodulatory effects. Pegylated interferon (PEG-IFN) remains an important first-line treatment option for CHB, especially in hepatitis B e antigen (HBeAg)-positive CHB, because a long-term off-treatment sustained response can be achieved in about 25% of patients after a finite treatment course [4–6]. Recent international guidelines highlight that seroconversion of (HBeAg) is a surrogate endpoint that is considered to be a marker for durable therapeutic response and improved clinical outcome in HBeAg-positive patients with chronic hepatitis B [3, 7–9]. Additionally, patients who achieve HBeAg seroconversion have an increased chance of clearing HBsAg during long-term follow-up [10, 11]. However, only part of HBeAg-positive CHB patients achieve HBeAg seroconversion at 24 weeks posttreatment. [4, 6] Early identification of responders would be of considerable benefit to clinicians, as it would allow therapy to be initiated only in patients likely to achieve a response, and to be modified in those patients unlikely to respond to the standard duration (48 weeks) of PEG-IFN monotherapy [5, 12–15].

Early studies on interferon-based therapies for chronic hepatitis B identified several factors that were associated with a higher likelihood of HBeAg seroconversion, including elevated serum alanine aminotransferase activity, lower levels of hepatitis B virus deoxyribonucleic acid (HBV DNA), and increased histologic activity in biopsy specimens [3, 16–19]. In addition, there is increasing interest in the association between HBV RNA level and response of patients treated with interferon and Polymerase Inhibitors [20–23]^.^

During viral morphogenesis, pregenomic ribonucleic acid (pgRNA) is encapsidated into core particles and reverse transcribed by HBV DNA polymerase into relaxed circular DNA (rcDNA). Mature rcDNA-containing virions are then enveloped and released from infected hepatocytes [1, 24, 25]. Importantly, in addition to HBV DNA, hepatitis B virus ribonucleic acid (HBV RNA) has also been detected in the serum of chronic hepatitis B (CHB) patient [22, 26, 27]. A possible association between serum HBV RNA levels and the presence of HBeAg was postulated in a previous study [27]. Recent studies revealed that a lower baseline plasma HBV RNA level was independently associated with response to PEG-IFN [23]. These potential relationship prompted us to carry out a detailed investigation into serum HBV RNA levels during treatment with PEG-IFN.

## Methods

### Aim

Here, we studied whether levels of serum HBV RNA might be helpful for predicting HBeAg seroconversion during treatment of PEG-IFN alfa-2a.

### Study participants

**Fig. 1:**
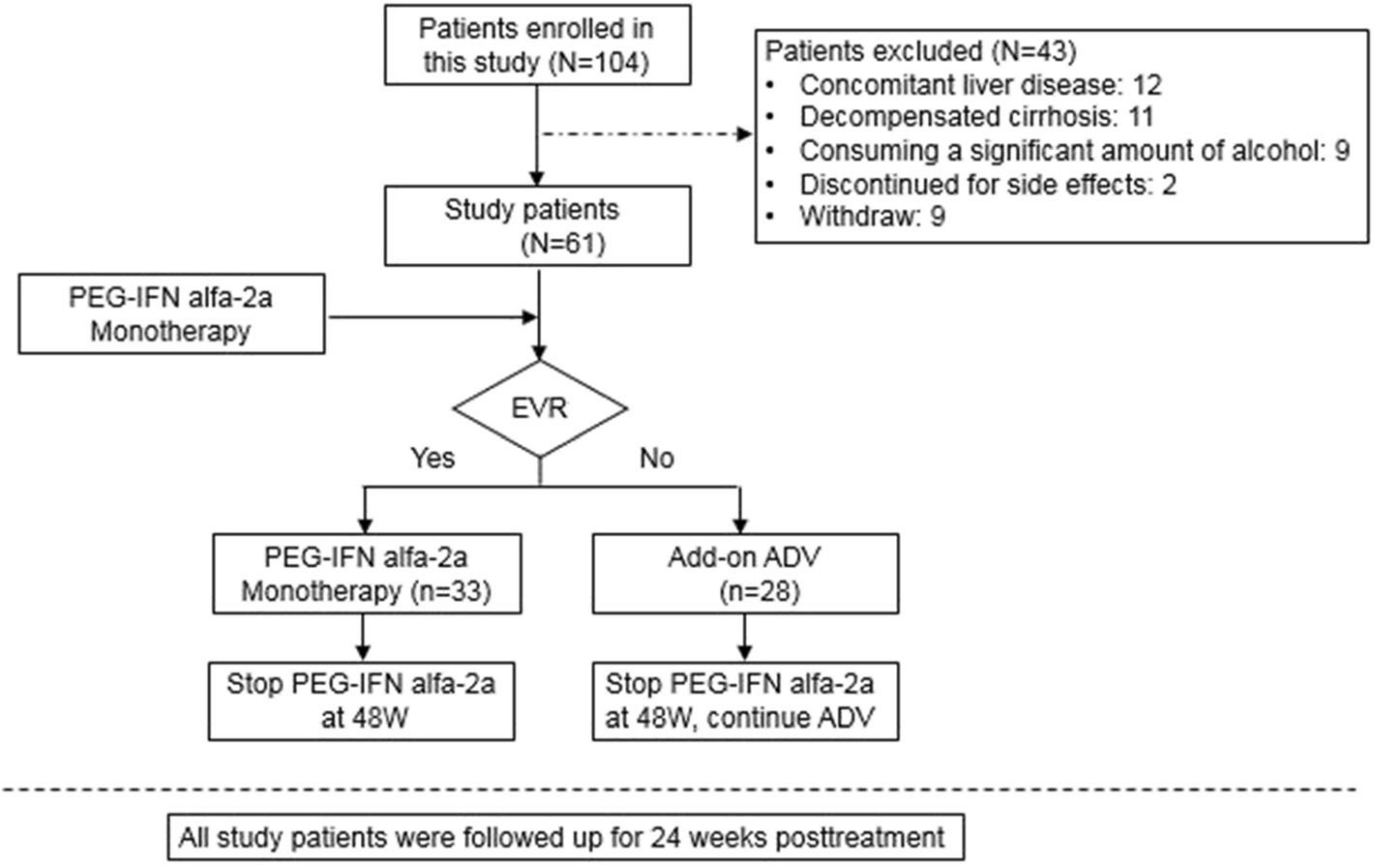
Flow chart of the study population and design. EVR: EVR was defined as HBV DNA decrease to < 100,000 copies/ml at week 12; ADV: adefovir; EVR: early virological response; PEG-IFN alfa-2a: Peginterferon alfa-2a

A total of 104 patients with chronic HBV infection receiving PEG-IFN alfa-2a (180 μg/week) alone or in combination with adefovir (ADV) were enrolled in this retrospective study. Patients were recruited from Huashan Hospital, Fudan University from January 2012 to December 2015. The inclusion criteria were pre-treatment HBsAg positive for > 6 months, HBeAg positive for > 6 months, no exposure to NAs or interferon (including conventional and pegylated forms) within the 6 months. Patients with concomitant liver diseases including chronic hepatitis C or D infection, Wilson’s disease, autoimmune hepatitis, primary biliary cirrhosis, decompensated cirrhosis, or those consuming a significant amount of alcohol (30 g/day for men, 20 g/day for women) were excluded. Patients were also excluded if they had evidence of anemia (hemoglobin levels < 11.5 g/dL, for women and < 12.5 g/dL for men), a neutrophil count < 1500 cells/mm3, or a platelet count < 90,000 cells/mm^3^ at screening. In total 61 patients who met the inclusion criteria were enrolled (Fig. 1).

The study was approved by the ethical committee of Jing’An District Centre Hospital of Shanghai (2017–18). All subjects gave informed consent for their participation in the study.

### Study design

The protocol is shown in Fig. 1. Each patient started with PEG-IFN alfa-2a monotherapy. The first period proposed for monitoring was based on early virological response (EVR; HBV DNA decrease to < 10,000 copies/ml at week 12). We made the decision to add ADV at week 12, based on 12 week EVR. Patients who did not meet 12 week EVR required ADV add-on at week 12. Subsequently, patients who treated with PEG-IFN alfa-2a monotherapy stopped PEG-IFN alfa-2a at week 48. However, those who add-on ADV stopped PEG-IFN alfa-2a administration at week 48, but continued oral ADV therapy. HBeAg, and antibody to the hepatitis B e antigen (anti-HBe), HBsAg, HBV DNA, HBV RNA were analyzed at baseline, during therapy at weeks 24, 48 and 24 weeks posttreatment. Patients who exhibited HBeAg seroconversion were regarded as responders, whereas those who did not achieve seroconversion were considered non-responders.

### Safety

We assessed the frequency, nature, and severity of adverse events, as well as changes in clinical laboratory parameters and vital signs from baseline. Adverse events and concomitant medications were recorded every 4 weeks. We analyzed adverse events according to the World Health Organization recommendations for toxicity grading, as adapted for chronic liver disease.

### Clinical and standard laboratory assessment

Relevant clinical variables included age, sex, serum alanine aminotransferase (ALT), aspartate aminotransferase (AST) levels. ALT, AST were measured in UniCel Dxc 800 using Synchron system Kits (Beckman Coulter). Serum HBV DNA concentration was assessed by real-time polymerase chain reaction with a lower detection limit of 500 copies/mL (DAAN Diagnostics, Guangzhou, China). Serum HBsAg, HBeAg, and antibody to the hepatitis B e antigen (anti-HBe) were measured using commercially available immunoassays (Abbott Laboratories, Chicago, IL). Serum HBsAg level’s detection value ranges from 0.05 to 250 IU/mL, and samples with an HBsAg level > 250 IU/mL required a 1:500 or greater dilution. All the test above are subject to strict quality control. Furthermore, our laboratory has passed the certificate from the College of American Pathologists (CAP).

### Quantification of HBV RNAs

Total nucleic acids were isolated from 200 μL serum using the High Pure Viral Nucleic Acid Kit (Roche Diagnostics) following the manufacturer’s protocol and Quantification of HBV RNAs (Roche Diagnostics). Isolated HBV RNA was reverse transcribed using Quantscript RT Kits (Tiangen Biotech, Beijing, China) with Olige (dT). For detection of the resulting HBV cDNAs, the real-time PCR analyses were performed (ABI Prism 7300 Sequence Detection System (Applied Biosystems, Foster City, CA). A 25-lL volume of reaction mixture containing Taqmen PCR Master Mix (Applied Biosystems), 200 nM of forward primer (5′-GGTCCCCTAGAAGAAGAACTCCCT-3′, nucleotides nt 2361–2384), 200 nM of reverse primer (CATTGAGTTCCCGAGATTGAGAT, nucleotides nt 2425–2448), Taqmen Probe (5′-FAM-TCTCAATCGCCGCGTCGCAGA-TAMRA-3′, nucleotides nt 2402–2422) and 2 uL of cDNA solution was prepared. Amplification was performed after an activation step at 95 °C for 1 min with 40 two-step cycles of 15 s at 95 °C and of 30 s at 60 °C ending with a cooling step down to 4 °C. In parallel, one negative control, one positive control, and a re-calibrator were analyzed. Dilution series of plasmid-based positive controls were used to generate external calibration curves for quantitative analysis. The lower limit of detection was 500 copies/mL.

### Statistical analysis

All data were processed using Stata 10.0 software (Stata Corporation, College Station, TX, USA). Categorical variables are expressed as number (%), and quantitative variables are shown as mean ± standard deviation for paired data or as median (Range) for unpaired data. Chi-square or Fisher’s exact test was used to compare categorical variables, while for quantitative variables the t test or Mann-Whitney’s test (unpaired data) or the t test or Wilcoxon’s test (paired data) were used. Spearman’s correlation coefficient (r) was used for correlation analysis. Additionally, Receiver operating characteristic curves were generated to compare the relative sensitivity and specificity of HBsAg, HBV DNA and HBV RNA as a predictor of HBeAg seroconversion. The cut off value was chosen according to the receiver operating characteristic curve when the sensitivity and specificity were both relatively high for the selective baseline factor. All tests were two-sided and used a significance level of 0.05.

### Ethical approval

The study was approved by the ethical committee of Jing’An District Centre Hospital of Shanghai (2017–18). All subjects gave informed consent for their participation in the study.

## Results

### Patient baseline clinical characteristics

The baseline characteristics of the 61 HBeAg-positive CHB patients are shown in Table 1. The mean age was 34.0 ± 8.0 years (range, 23–57), and 75% of them were male. The mean value of serum HBV DNA level and HBV RNA level were 6.72 ± 1.91 log_10_ copies/mL and 5.37 ± 1.32 log_10_ copies/mL, respectively. The mean value of serum HBsAg level was 3.89 ± 0.71 log_10_ IU/mL. The ALT and AST values were 120.0 IU/mL (range 13–603 IU/mL) and 89.0 IU/mL (range 21–435 IU/mL), respectively. HBV RNA levels were strongly correlated with HBV DNA and HBsAg levels. There was a strong inverse correlation between serum HBV RNA and HBV DNA (HBV RNA vs HBV DNA: r = 0.684, *P* = 0.004, HBV RNA vs HBsAg: r = 0.521, *P* < 0.001) (Supple).

**Table 1:** Baseline characteristics of responders and non-responders

Variables	Overall populations	Responders	Non-responders	*P* value
Number	61	22	39	–
Age (year)	33.0 (23–57)	31.0 (23–57)	36.5 (23–57)	0.043
Male(%)	46 (75)	16 (73)	30 (77)	0.493
ALT (IU/L)	120.0 (13–603)	175.0 (43–575)	88.0 (13–603)	0.025
AST (IU/L)	89.0 (21–435)	140.0 (36–435)	68.0 (21–399)	0.031
HBV RNA (log_10_ copies/mL)	5.37 ± 1.32	4.55 ± 1.19	5.90 ± 1.13	0.001
HBV DNA (log_10_ copies/mL)	6.72 ± 1.91	5.76 ± 2.24	7.15 ± 1.59	0.036
HBsAg (log_10_ IU/mL)	3.89 ± 0.71	3.25 ± 0.63	4.24 ± 0.53	< 0.001

*ALT* alanine aminotransferase, *AST* aspartate aminotransferase, *HBsAg* hepatitis B surface antigen, *HBV DNA* hepatitis B deoxyribonucleic acid, *HBV RNA* hepatitis B ribonucleic acid

Data were expressed as mean values± standard deviation or median (range)

### Relationship between HBeAg seroconversion and baseline serum HBV RNA levels, other serum markers of HBV replication

Of the 61 patients, 22 patients (36.1%) exhibited HBeAg seroconversion at 24 weeks posttreatment (responders), and 39 (52%) were non-responders. In terms of the probability of HBeAg seroconversion, there was no statistically significant difference between the PEG-IFNa2a alone and combination with adefovir (ADV combine with PEG-IFNa2a vs monotherapy: 9/28 vs 13/33, *P* = 0.602). Baseline serum HBV RNA level for the responses and non-responsers were 4.55 ± 1.19 log_10_ copies/mL (range 2.70–6.94 log_10_ copies/mL) and 5.90 ± 1.13 (range 3.10–7.52 log_10_ copies/mL) (*P* = 0.001), respectively. Baseline serum HBV RNA levels were significantly lower for responses compared to non-responses. Furthermore, the baseline HBV DNA level and HBsAg level were significantly lower in responders than in non-responders (All *P* < 0.05, Table 1). Additional, responsers were of younger age, higher ALT and AST levels (All *P* < 0.05, Table 1). The percentage of men was similar between responders and non-responders.

### Receiver operating characteristics curves: prediction of HBeAg seroconversion

**Fig. 2:**
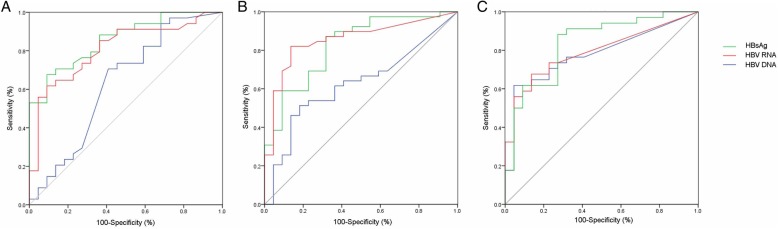
Receiver operating characteristic curves predicting HBeAg seroconversion through posttreatment follow-up. Receiver operating characteristic curves for HBV RNA (red line), HBV DNA (blue line) and HBsAg (green line) as they predict HBeAg seroconversion, using data derived from their respective serum values at baseline, week 12, 24. **a**. Baseline data predict HBeAg seroconversion: Area under curve (AUC): HBV RNA 0.810; HBV DNA 0.630; HBsAg: 0.816; **b**. Week 12 data predict HBeAg seroconversion: AUC: HBV RNA 0.854; HBV DNA 0.678; HBsAg: 0.825; **c**. Week 24 data predict HBeAg seroconversion: HBV RNA 0.817; HBV DNA 0.779; HBsAg: 0.824

Overall, HBV RNA, HBV DNA and HBsAg levels decreased consistently during treatment, we investigated the discriminatory capabilities of HBV RNA decline at baseline, weeks 12 and 24 for predicting response. Using receiver operating characteristic curve analysis, AUCs were 0.810, 0.854 and 0.817 for baseline, week 12 and 24, respectively. For predicting response at 24 weeks posttreatment, the discriminatory values of absolute HBsAg and HBV DNA levels were also investigated. When balancing sensitivity and specificity, HBsAg is the best predictor of HBeAg seroconversion at baseline and week 24 while HBV RNA is the best predictor at week 12 (Fig. 2).

**Fig. 3:**
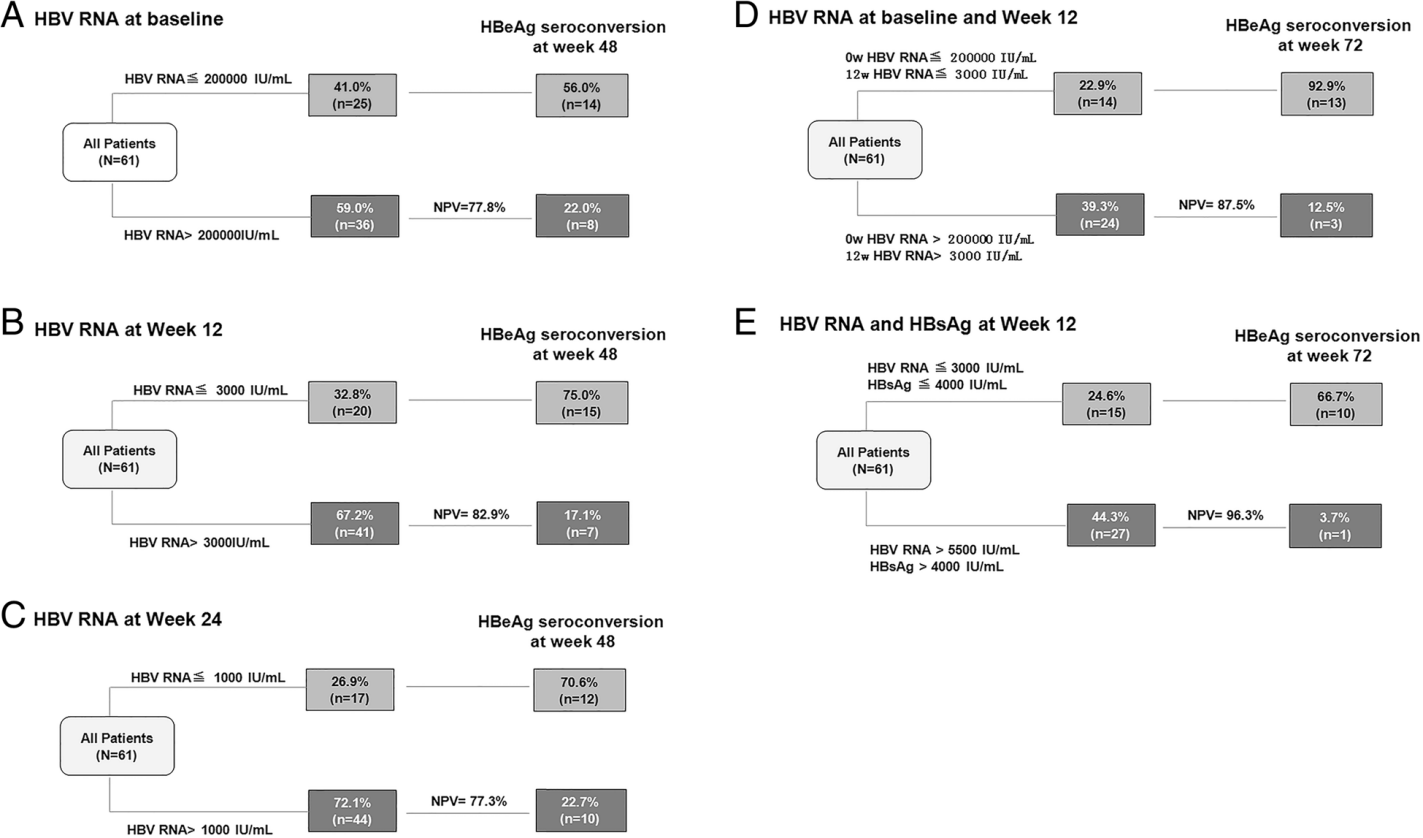
**a**: Quantitative HBV RNA at baseline yields a high NPV for HBeAg seroconversion. Analysis yielded an NPV of 77.8% for HBV RNA > 200,000 copies/mL at baseline; **b**: Positive predictive value (PPV) of HBV RNA at week 12 as a predictor of HBeAg seroconversion. Analysis yielded a PPV of 75.0% for HBV RNA ≤ 3000 copies/mL, of whom 17.1% achieved HBeAg seroconversion yielding a NPV of 82.9%. **c**: Serum HBV RNA at week 24 yielded a PPV of 70.6% and NPV of 77.3% for predicting HBeAg seroconversion. **d**: Serum HBV RNA at baseline and week 12 of treatment relationship to HBeAg seroconversion: Among patients with HBV RNA ≦ 200,000 copies/mL at baseline and ≦ 3000 copies/mL at week 12, 92.9% achieved a response. However, the rates of response among patients with HBV RNA > 200,000 copies/mL at baseline and > 3000 copies/mL at week 12, only 12.5%. HBV RNA, hepatitis B ribonucleic acid (copies/mL). **e**: Among patients with HBV RNA ≦ 3000 copies/mL and HBsAg ≦ 4000 IU/mL at week 12, 66.7% achieved a response. Whereas, the rates of response among patients with HBV RNA > 3000 copies/mL and HBsAg ≦ 4000 IU/ Ml at week 12, only 3.7% patients achieved a response. HBV RNA, hepatitis B ribonucleic acid (copies/mL)

Next, Youden index was proceeded to investigate the optimal cutoff point. The optimal cutoff level of serum HBV RNA to predict response were 200,000, 3000 and 1000 copies/mL at baseline, week 12 and 24 respectively. Response rates and negative predictive value (NPV) were calculated for response at 24 weeks posttreatment (Table 2, Fig. 2). At baseline, 56.0% of patients with HBV RNA ≦ 200,000 copies/mL attained seroconversion. On the other hand, of who with HBV RNA >  200,000 copies/mL, only 22.2% achieved a response. Consequently, the NPV of HBV RNA ≦ 200,000 copies/mL is 77.8% for prediction of response (Table 2 Fig. 3a). At week 12, the PPV of HBV RNA ≦ 3000 copies/mL was 75.0% for prediction of response (Table 2 Fig. 3b). Furthermore, PPV and NPV of HBV RNA ≦1000 copies/mL at week 24 was 70.6 and 77.3%, respectively (Table 2 Fig. 3c). Moreover, we examined the rates of response among patients with HBV RNA ≦ 200,000 copies/mL at baseline and ≦ 3000 copies/mL at week 12. The results showed that among the above subgroups, 92.9% achieved a response at 24 weeks posttreatment (Fig. 3d).

**Table 2:** Levels of serum HBV RNA at baseline and on-treatment: relationship to response at 24 weeks posttreatment

	HBV RNA (copies/mL)	Total No. of patients	No. of patients with HBeAg seroconversion	Percentage of patients with HBeAg seroconversion
Baseline	≦ 200,000	25	14	56.0%
	> 200,000	36	8	22.2%
Week 12	≦ 3000	20	15	75.0%
	> 3000	41	7	17.1%
Week 24	≦ 1000	17	12	70.6%
	> 1000	44	10	22.7%

*HBV RNA* hepatitis B ribonucleic acid

The optimal cutoff level of serum HBsAg to predict response were 8000, 4000 and 2000 IU/mL at baseline, week 12 and 24 respectively. When we combine HBV RNA >  3000 copies/mL with HBsAg > 4000 IU/mL at week 12, the results showed that only 3.7% patients achived HBeAg seroconversion (NPV 96.3%) (Fig. 3e, Additional file 1: Table S1).

### Factors associated with HBeAg seroconversion at 24 weeks posttreatment

Younger age, lower level of HBV RNA and HBsAg at baseline, lower level of HBV RNA and HBsAg at week 12, lower level of HBV DNA, HBV RNA, HBsAg at week 24 (all *P* < 0.05) were correlated with response at 24 weeks posttreatment by univariate analysis. After adjusting for different clinical parameters, factors independently associated with response at 24 weeks posttreatment included younger age (OR = 0.687, *P* = 0.040), ALT×ULN at baseline (OR = 1.660, *P* = 0.041), Baseline HBV RNA ≦ 200,000 copies/mL (OR = 1.841, *P* = 0.008), Baseline HBsAg ≦ 8000 IU/mL (OR = 3.791, *P* = 0.003), HBV RNA ≦ 3000 copies/mL at week 12 (OR = 12.458, *P* = 0.039) and HBsAg ≦ 2000 IU/mL at week 24 (OR = 3.297, *P* = 0.031) (Table 3).

**Table 3:** Multivariate and univariate analyses of factors associated with HBeAg seroconversion at 24 weeks posttreatment

Variables	Univariate analysis			Multivariate analysis		
	Odd ratio	95% confidence interval	*P* value	Odd ratio	95% confidence interval	*P* value
Sex (male)	0.810	(0.241–2.651)	0.715			
Age (years)	0.896	(0.818–0.981)	0.018	0.687	(0.384–0.850)	0.040
ALT (U/L) × ULN at baseline	1.315	(0.977–1.768)	0.071	1.660	(1.097–2.875)	0.041
AST (U/L) × ULN at baseline	1.298	(0.849–1.501)	0.087			
Baseline HBV DNA ≤ 30,000,000 copies/mL copies/mL	1.636	(0.542–4.937)	0.382			
Baseline HBV RNA ≤ 200,000 copies/mL	3.818	(1.228–12.871)	0.021	1.841	(1.175–3.708)	0.008
Baseline HBsAg ≤8000 IU/mL	6.001	(1.787–20.150)	0.004	3.791	(1.969–25.358)	0.003
HBV DNA ≤ 200,000 copies/mL at week 12	3.429	(0.969–12.131)	0.056			
HBV RNA ≤ 3000 copies/mL at week 12	5.357	(1.605–17.879)	0.006	12.458	(3.969–80.494)	0.039
HBsAg ≤4000 IU/mL at week 12	7.363	(2.150–25.223)	0.001			
HBV DNA ≤ 30,000 copies/mL at week 24	12.681	(1.783–60.873)	0.012			
HBV RNA ≤ 1000 copies/mL at week 24	6.500	(1.915–22.051)	0.003			
HBsAg ≤2000 IU/mL at week 24	7.519	(2.232–25.316)	0.001	3.297	(1.346–21.391)	0.031

*ALT* alanine aminotransferase, *AST* aspartate aminotransferase, *HBsAg* hepatitis B surface antigen, *HBV DNA* hepatitis B deoxyribonucleic acid, *HBV RNA* hepatitis B ribonucleic acid

### Relationship between sustained HBV DNA virological responders and HBV RNA

**Fig. 4:**
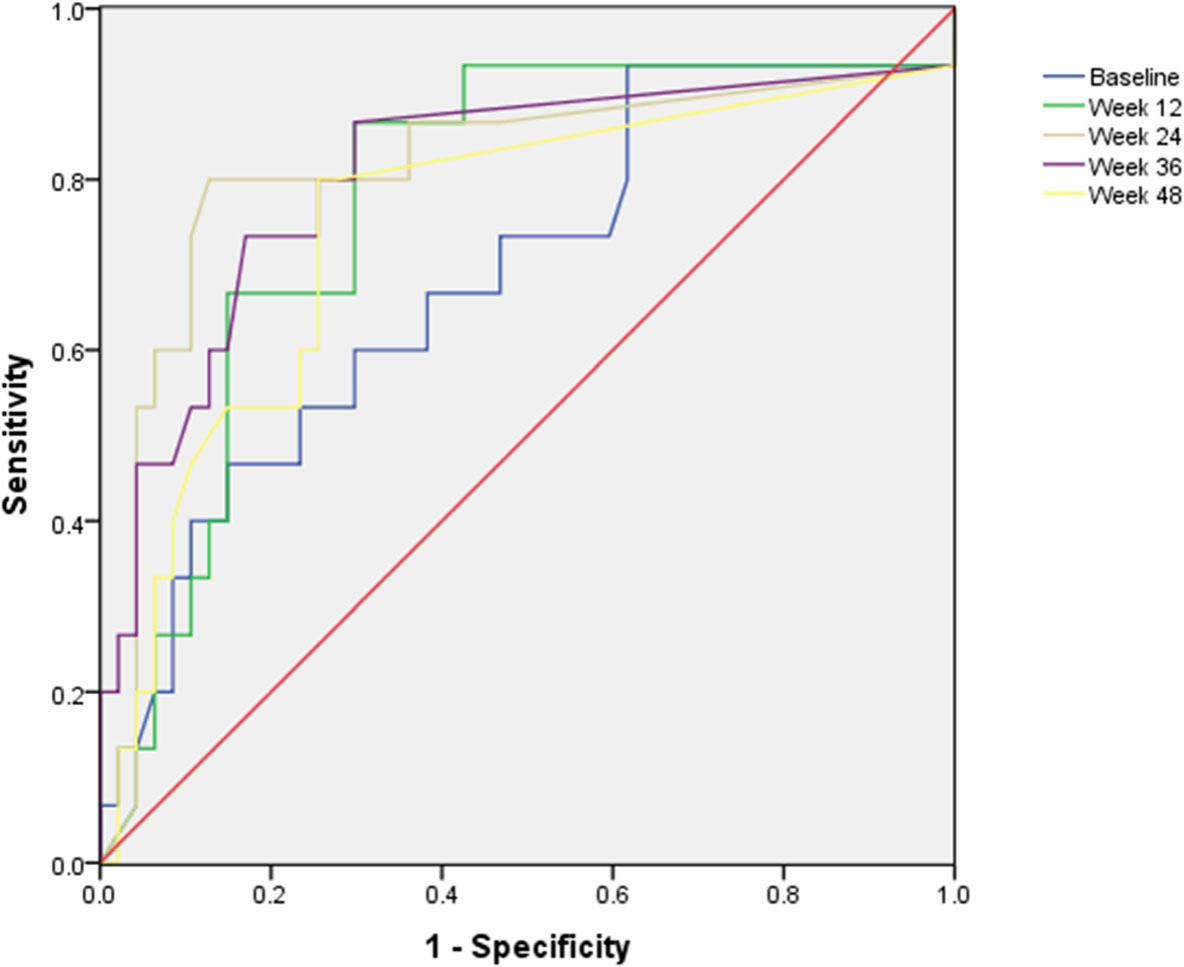
Receiver operating characteristic curves predicting sustained virological response. Receiver operating characteristic curves for HBV RNA at baseline (blue line), HBV RNA at week 12 (green line), HBV RNA at week 24 (grey), HBV RNA at week 36 (purple), HBV RNA at week 48 (yellow line) as they predict sustained virological response. AUC: HBV RNA at baseline 0.685; HBV RNA at week 12 0.777; HBV RNA at week 24 0.797; HBV RNA at week 36 0.766, HBV RNA at week 48 0.747

Sustained serum HBV DNA response, as well as HBeAg seroconversion in HBeAg-positive CHB patients are the desired treatment endpoints. In our study, patients who exhibited HBV DNA < 10,000 copies/mL at week 48 and 24 weeks posttreatment were regarded as virological responders (VR), whereas those who did not achieve it were considered non-VR. Baseline serum HBV RNA levels were significantly lower for virological response (VR) compared to non-VR (*P* = 0.013). Similarly, on treatment serum HBV RNA levels were significantly lower for VR compared to non-VR (all *P* < 0.05) (Table 4). Using receiver operating characteristic curve analysis for predicting VR, HBV RNA at week 24 is the best predictor (AUC 0.797, Standard Error 0.079, *P* < 0.001) (Fig. 4). The optimal cutoff level of serum HBV RNA at week 24 to predict VR were Log_10_ 3.76, the nearest two thousand level is 5000 copies/mL, yielded a PPV of 68.1% for HBV RNA ≤ 5000 copies/mL, of whom 29.5% achieved VR yielding a NPV of 70.5%.

**Table 4:** HBV RNA levels in virological responders and non virological responders

Variables	VR	Non-VR	*P* value
Number	42	19	–
Baseline HBV RNA (log_10_ copies/mL)	4.81 ± 1.39	6.20 ± 0.80	0.013
Week 12 HBV RNA (log_10_ copies/mL)	3.35 ± 0.80	4.93 ± 0.86	< 0.001
Week 24 HBV RNA (log_10_ copies/mL)	3.04 ± 0.67	4.49 ± 1.04	< 0.001
Week 36 HBV RNA (log_10_ copies/mL)	2.79 ± 0.21	3.65 ± 0.75	< 0.001
Week 48 HBV RNA (log_10_ copies/mL)	2.84 ± 0.40	3.15 ± 0.47	0.011

*HBV RNA* hepatitis B ribonucleic acid, *VR* patients who achieved sustain virological response, non-VR, patients who didn’t achieve sustain virological response

Data were expressed as mean values± standard deviation

## Discussion

Nowadays, more and more doctors are taking the initiative in individualized treatment for chronic hepatitis B patients. With the purpose of taking individualized treatment, it is important to evaluate the baseline status of each patient at the start of treatment and to then decide which antiviral drug is the best choice. For those patients who are not likely to benefit from PEG-IFN therapy, an early switch to NA is essential. Our intention was to find a new predictive marker for serologic response in HBeAg positive CHB patients during treated with Peg-IFN alfa-2a. In the present study we have analyzed the level of HBV RNA in serial serum samples derived from chronically HBV infected patients receiving Peg-IFN. Then, we focused on baseline and on-treatment predictors for HBeAg seroconversion at 24 weeks posttreatment week.

Previous study confirmed that the HBV RNA detected in serum is pgRNA. We set up a method to quantify HBV RNA in serum specimens by using a specific primers which carries the polyadenylation signal downstream of the HBx open reading frame (ORF) at position nt 2361. HBV RNA were all detectable from all serum of baseline, which were similar from the study before [23].

The results showed baseline HBV RNA levels were strongly correlated with HBV DNA and HBsAg levels in untreated patients (HBV RNA vs HBV DNA: r = 0.684, *P* = 0.004, HBV RNA vs HBsAg: r = 0.521, *P* < 0.001), an observation which was also previously reported supporting the hypothesis that polyadenylated serum HBV RNA is a marker of HBV replication [21, 23, 28].

We observed that baseline HBV RNA levels were lower in patients achieving response than in non-responders (4.55 ± 1.19 and 5.90 ± 1.13 copies/mL, respectively, *P* = 0.001). Moreover, Baseline HBV RNA cut off level (200,000 copies/mL) provided a positive predictive value (PPV) of 56.0% and a negative predictive value (NPV) of 77.8% for response. In additional, baseline HBV RNA ≦ 200,000 copies/mL was an independent factor associated with response (OR 1.841, *P* = 0.008). The results were similar with the Florian’s research [29]. Younger age, higher level of ALT, lower level of HBsAg and HBV DNA at baseline were independently related to response as expected, which were similar with previous studies [5, 6, 30, 31].

In line with these observations was the stronger decline in HBV RNA levels in responsers after treatment with non-responders. ROC curves showed that AUC of HBV RNA (0.854) was superior to that of HBsAg (0.825) in predicting response at week 12. What was consistently to the study before [29].

Furthermore, we examined the rates of response among patients with HBV RNA >  200,000 copies/mL at baseline and >  3000 copies/mL at week 12. The results showed that among the above subgroups, only 12.5% (3/24) achieved a response (NPV 87.5%) (Fig. 3d). This findings indicate that prediction of nonresponse to PEG-IFN alfa-2a is possible as early as week 12 and comparable to HBsAg monitoring [31]. The factors affecting HBeAg seroconversion 24 weeks posttreatment were analyzed in our study. Meanwhile, only 3.7% patients with HBV RNA > 3000 copies/mL with HBsAg > 4000 IU/mL at week 12 achived HBeAg seroconversion. The results advised the subgroup should change the treatment.

The factors affecting HBeAg seroconversion at 24 weeks posttreatment were analyzed in our study. Multivariate analysis showed that HBV RNA < 200,000 copies/mL at baseline, HBV RNA < 3000 copies/mL at week 12 were independent predictors for HBeAg seroconversion at 24 weeks post-PEG-IFN treatment, with the latter being the strongest predictor (OR = 12.458 *P* = 0.039). Furthermore, younger age, ALT×ULN at baseline, HBsAg at baseline, HBsAg at week 24 were independent predictors for HBeAg seroconversion at 24 weeks posttreatment other than HBV RNA. These findings were consistent with the research before [13, 30, 32].

Sustained virological response in HBeAg-positive CHB patients are the desired treatment endpoints. Sustained virological response after Peg-IFNa is usually associated with remission of the liver disease. In our present study, HBV RNA levels were significantly lower, at all time points for virological response (VR) compared to non-VR (all *P* < 0.05). AUC of HBV RNA at week 24 is 0.797, showing PPV 68.1% and NPV 70.5% for 5000 copies/mL. For the reason that risk of HBV reactivation seems to been there for years. All such patients require long-term follow-up because of the risk of exacerbation with development of HBeAg negative CHB or even of HBeAg seroconversion in initially HBeAg positive patients [33]. At the same time, the patients only follow-up for 24 weeks posttreatment in our study. Thus, further studies with long term follow-up are needed to proceed.

Interest in HBV RNA as a marker of sustained response to interferon-based therapy has been based upon studies that showed a positive association between HBV RNA and covalently closed circular DNA (cccDNA) [34–36]. CccDNA reflects the number of hepatocytes infected with the virus and acts as a template for transcription of viral genes [37–39]. The ongoing presence of cccDNA in hepatocytes even when serum HBV DNA levels are undetectable is responsible for the persistence and potential recurrence of HBV infection [40]. The immunomodulatory activity of interferon results in activation of cytotoxic T cells [41]. These cells are important in clearing infected hepatocytes and thereby act to reduce levels of cccDNA [42]. Taken together, these data suggest that HBV RNA level is an appropriate way to monitor the ongoing immune clearance of infected hepatocytes and elimination of cccDNA mediated by PEG-IFN alfa-2a. Interestingly enough, serum HBV RNA may better reflect the activity of intrahepatic cccDNA than serum HBV DNA during NA treatment. It is based on the fact that formation of rcDNA is blocked by NA. However, HBV RNA virion-like particle is not be affected [22, 43, 44]. Thus, HBV RNA has been found to be associated with HBeAg seroconversion [21, 45], response to NA treatment [43, 46], the safe of discontinue of NA treatment [22, 47], the emergence of mutant [48].

It is advised that HBV RNA level in serum could be used as a new tool for predicting response to PEG-IFN alfa-2a treatment in HBeAg-positive patients. The prediction of nonresponse to PEG-IFN alfa-2a is possible as early as week 12. According to our results, baseline HBV RNA levels combined with week 12 could further improve the predictive value for predicting response. Nonetheless, information on HBV genotypes was not available and the limited number of patients achieving HBeAg seroconversion included in our study represents a limitation. Therefore further studies on serum HBV RNA as a response marker are needed to confirm these findings.

## Conclusions

In conclusion, we showed that HBV RNA is detectable in serum specimens. A 48-week course of PEG-IFN alfa-2a results in a significant decline in serum HBV RNA in patients with HBeAg-positive CHB. Patients who with HBV RNA > 200,000 copies/mL at baseline and > 3000 copies/mL at week 12 have a low chance of achieving HBeAg seroconversion 24 weeks posttreatment, and should therefore be considered for treatment discontinuation. Meanwhile, patients with HBV RNA > 3000 copies/mL and HBsAg > 4000 IU/mL at week 12 should change the treatment as well.

## Additional file

Additional file 1: **Figure S1.** Scatter plots showing levels of HBV RNA and HBsAg. HBV RNA and HBV DNA (both in log_10_ copies/mL) before PEG-IFN alfa-2a treatment in 61 HBeAg positive patients. A. The level of HBV RNA was significantly correlated with HBV DNA before treatment (r = 0.684, *P* = 0.004); B. The level of HBV RNA was significantly correlated with HBsAg before treatment (r = 0.521, *P* < 0.001). *r*: Pearson’s correlation coefficient; *P*: *p* value of the correlation t-test. **Table S1.** Levels of serum HBV RNA, HBsAg, HBV DNA relationship to response at 24 weeks posttreatment. (DOCX 129 kb)

## Abbreviations

ADV: Adefovir
CHB: Chronic hepatitis B
EVR: Early virological response
HBeAg: Hepatitis B e antigen
HBV DNA: Hepatitis B virus deoxyribonucleic acid
HBV RNA: Hepatitis B virus ribonucleic acid
HBV RNA: Hepatitis B virus ribonucleic acid
HBV: hepatitis B virus
HCC: Hepatocellular carcinoma
NA: nucleos(t)ide analogues
PEG-IFN: Pegylated interferon
pgRNA: Pregenomic ribonucleic acid
rcDNA: Relaxed circular DNA
