# Supplementary material for: Serum hepatitis B virus RNA levels as a predictor of HBeAg seroconversion during treatment with peginterferon alfa-2a
Source: Virol J. 2019 May 7;16:61. doi: 10.1186/s12985-019-1152-6 (PMC6505123; doi:10.1186/s12985-019-1152-6)
Supplement: Supplementary file 1 — Figure S1. Scatter plots showing levels of HBV RNA and HBsAg. HBV RNA and HBV DNA (both in log10 copies/mL) before PEG-IFN alfa-2a treatment in 61 HBeAg positive patients. A. The level of HBV RNA was significantly correlated with HBV DNA before treatment (r = 0.684, P = 0.004); B. The level of HBV RNA was significantly correlated with HBsAg before treatment (r = 0.521, P < 0.001). r: Pearson’s correlation coefficient; P: p value of the correlation t-test. Table S1. Levels of serum HBV RNA, HBsAg, HBV DNA relationship to response at 24 weeks posttreatment. (DOCX 129 kb) [file 12985_2019_1152_MOESM1_ESM.docx]

**Figure S1**

**
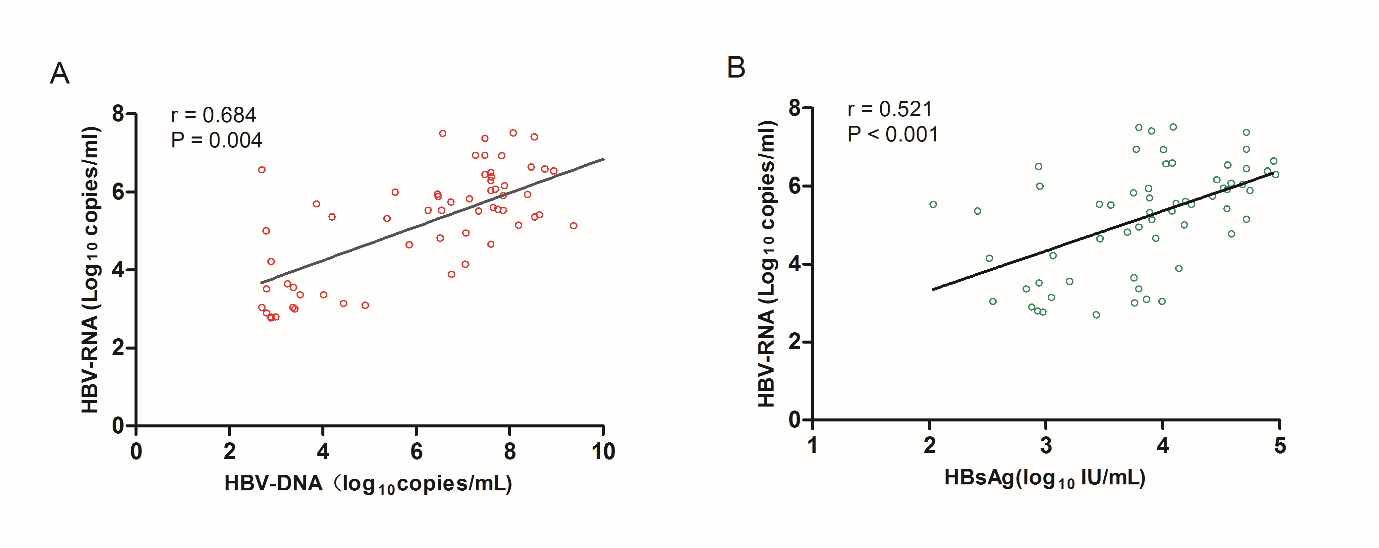
**

Scatter plots showing levels of HBV RNA (log_10_ copies/mL) and HBsAg (log_10_ IU/mL): HBV RNA and HBV DNA (both in log_10_ copies/mL) before PEG-IFN alfa-2a treatment in 61 HBeAg positive patients. A. The level of HBV RNA was significantly correlated with HBV DNA before treatment (r = 0.684, *P* = 0.004); B. The level of HBV RNA was significantly correlated with HBsAg before treatment (r = 0.521, *P* < 0.001). r: Pearson’s correlation coefficient; *P*: p value of the correlation t-test.

|  | AUC | Cut off (Log) | Nearest two thousand level | Standard error | P value | 95% CI | PPV | NPV |
| --- | --- | --- | --- | --- | --- | --- | --- | --- |
| HBV RNA (copies/mL) |  |  |  |  |  |  |  |  |
| Baseline | 0.810 | 5.30 | 200000 | 0.059 | <0.001 | 0.694-0.925 | 56.0% | 77.8% |
| Week 12 | 0.854 | 3.94 | 3000 | 0.050 | <0.001 | 0.754-0.950 | 75.0% | 82.9% |
| Week 24 | 0.817 | 3.01 | 1000 | 0.061 | <0.001 | 0.694-0.932 | 70.6% | 77.3% |
| HBV DNA (copies/mL) |  |  |  |  |  |  |  |  |
| Baseline | 0.630 | 7.47 | 30000000 | 0.074 | 0.100 | 0.481-0.770 | 53.3% | 66.3% |
| Week 12 | 0.678 | 5.28 | 200000 | 0.072 | 0.058 | 0.504-0.785 | 53.9% | 72.3% |
| Week 24 | 0.779 | 4.50 | 30000 | 0.062 | <0.001 | 0.641-0.885 | 57.2% | 74.8% |
| HBsAg (IU/mL) |  |  |  |  |  |  |  |  |
| Baseline | 0.816 | 3.96 | 8000 | 0.053 | <0.001 | 0.713-0.910 | 66.7% | 76.7% |
| Week 12 | 0.825 | 3.60 | 4000 | 0.053 | <0.001 | 0.723-0.931 | 68.1% | 84.6% |
| Week 24 | 0.824 | 3.20 | 2000 | 0.055 | <0.001 | 0.710-0.925 | 72.2% | 84.2% |

**Table S1** Levels of serum HBV RNA, HBsAg, HBV DNA relationship to response at 24 weeks posttreatment.

HBV RNA, hepatitis B [ribonucleic](javascript:void(0);) acid; HBsAg, hepatitis B surface antigen; HBV DNA, hepatitis B deoxyribonucleic acid; AUC, area under curve; CI, confidence interval; PPV, positive predictive value; NPV, negative predictive value.
